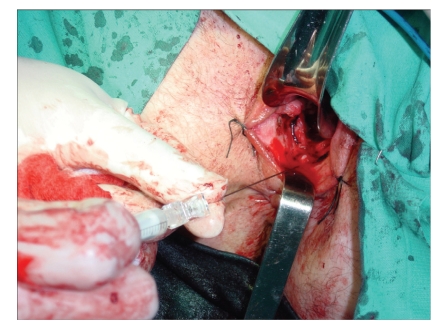# Adipose-Derived Stem Cells in Crohn's Rectovaginal Fistula

**DOI:** 10.1155/2010/961758

**Published:** 2010-03-07

**Authors:** D. García-Olmo, D. Herreros, P. De-La-Quintana, H. Guadalajara, J. Trébol, T. Georgiev-Hristov, M. García-Arranz

**Affiliations:** ^1^Department of Surgery, La Paz University Hospital-IdiPAZ, 28046 Madrid, Spain; ^2^Cell Therapy Laboratory, Foundation to Biomedical Research of La Paz University Hospital-IdiAZ, 28046 Madrid, Spain; ^3^School of Medicine, Autonomous University of Madrid, 28029 Madrid, Spain

## Abstract

Therapeutic options for recto-vaginal fistula in the setting of Crohn's disease are limited and many data are available in the literature. The manuscript describes the history of a patient who has been the pioneer of our Clinical Trials in treating this disease in fistulizing Crohn's disease environment. We believe it is the first time that a patient with this disease has been treated by adipose-derived stem cells in allogeneic form. The conclusion of our study with Mary is that the use of mesenchymal stem cells derived from adipose tissue is secure, either in autologous or allogeneic form. Furthermore, we have proved that if we use multi-dose and multiple applications on a patient, it does not produce any adverse effect, which confirms us the safety of using these cells in patients at least in the fistulizing Crohn's disease environment.

## 1. Introduction

Human Adipose-Derived Stem Cells emerge as key regulators of immune/inflammatory responses in vivo and as attractive candidates for cell-based therapies to treat IBD, sepsis and hence to improve healing [1]. To illustrate this sentence we believe that Mary's story (not her real name) could be a good IBD clinical picture that offers a glimmer of hope. 

The management of rectovaginal fistulas in patients with Crohn's disease continues to be extremely challenging and, indeed, somewhat frustrating [2]. Such fistulas are a very distressing complication that significantly reduces the quality of life of affected women. Various therapies have been proposed, such as advancement flap plasty [3], Martius plasty [4], gracilis transposition [5], and proctectomy and definitive colostomy, when a cure is impossible. It is also important to consider the incontinence rate associated to these procedures. In a study of 310 patients who underwent surgery (fistulotomy and rectal advancement flap) for anal incontinence, van Kooperen et al. [6] reported soiling in 40%, but there were no reports of anal incontinence associated with ASCs implantation. Recent improvements in medical treatment (e.g., infliximab) and expert surgical management have decreased the need for proctectomy. However, recurrence has a major negative impact on the quality of life. The suboptimal quality of perianal tissues that are affected by Crohn's disease is probably the origin of the failure to heal [4]. Long-term therapy with infliximab (as would be used in maintenance regimens) is generally well tolerated although clinicians are urged to be particularly vigilant for rare but serious adverse events such as serum sickness-like reaction, opportunistic infection and sepsis, and autoimmune disorders [7]. 

## 2. Case Presentation

**Figure 1:**
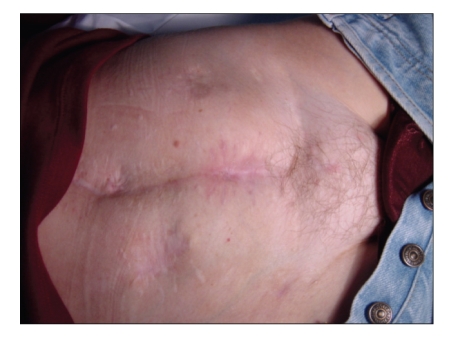


In 2002, we decided to test a cell-based therapeutic protocol on a young woman with Crohn's disease and recurrent intractable rectovaginal fistulas [8]. Autologous adipose-derived stem cells (ASCs) were chosen as the cell source because they are easily harvested using liposuction. Although Crohn's disease is the worst scenario in treatment of rectovaginal fistula, we observed satisfactory healing without fecal incontinence. In view of the successful outcome, a pilot study was started [9] and Mary, a 34-year-old woman diagnosed of Crohn's disease ten years before, was included. At the time, Mary had four enterocutaneous and one rectovaginal fistula. After liposuctions, hASCs were isolated, processed and expanded. The enterocutaneous fistulas healed after injection of hASCs according to our protocol (Figure 1). The rectovaginal fistula was also treated using hASCs (Figure 2), but complete healing was not achieved. 

Later, in 2004, we conducted a phase II clinical triala [10] that aimed to test the effectiveness of hASCs (investigational drug code: Cx401b) in the treatment of complex perianal fistula and Mary was once again included but assigned to the control group. A total of 8 women with rectovaginal fistulas participated (4 with Crohn's disease). Four women were treated with stem cells (treatment group) and complete closure was achieved in 3. The other 4 women—Mary included—were treated with fibrin glue (control group) with no healing in any of the cases. Mary's fistula therefore remained unhealed. 

During 2006 we designed two phase III clinical trials that aimed to definitively assess the efficacy of autologous ASCs in complex perianal fistula and these are currently underway. However, women with rectovaginal fistula were excluded to minimize clinical variability and so Mary was not eligible. We decided to treat her fistula by compassionate use according to the European regulatory laws and the Spanish Medicines Agency guidelines. After obtaining regulatory permission, a new liposuction procedure was performed and the protocol for Cx401 therapy started. Unfortunately, bacterial contamination occurred during the cell expansion process and treatment was aborted. To avoid further failure, after carefully consideration of the regulatory implications, we proposed a new attempt using, this time, allogenic ASCs. We obtained and processed adipose material from a donor and the ASCs obtained (investigational drug code: Cx601) were used to treat Mary's rectovaginal fistula. To our knowledge, this is the first time that allogenic ASCs have been used in humans. No rejection or adverse events were observed, but the fistula remained open. Nevertheless a great improvement was appreciated and a new cell injection is schedule. 

## 3. Discussion

This is Mary`s clinical picture so far. During her life, Mary will probably suffer further outbreaks of Crohn's disease that might produce new fistulas, but these could perhaps be treated once again with stem cells in an attempt to exploit de capacities of Adipose-Derived Stem Cells as regulators of inflammatory and healing responses.

## Figures and Tables

**Figure 2:**